# Intersection between Eating Disorders and Obesity in Youth: Implications for Treatment

**DOI:** 10.1007/s13679-025-00664-6

**Published:** 2025-10-14

**Authors:** Alyssa M. Button, Alicia Persaud, Anna B. Tanner, Katherine N. Balantekin, Marian Tanofsky-Kraff, Hiba Jebeile, Denise E. Wilfley

**Affiliations:** 1 Departments of Psychiatry & Pediatrics, Virginia Commonwealth University, Richmond, VA USA; 2 Public Health Sciences, Washington University in St. Louis, St. Louis, MO USA; 3 School of Medicine, Emory University, Atlanta, GA USA; 4 Exercise and Nutrition Sciences, University at Buffalo SUNY, Buffalo, NY USA; 5 Departments of Medical and Clinical Psychology & Medicine, Uniformed Services University of the Health Sciences, Bethesda, MD USA; 6 University of Sydney, Children’s Hospital Westmead Clinical School, NSW Sydney, Australia; 7 Psychological and Brain Sciences, Washington University in St. Louis, St. Louis, MO USA

**Keywords:** Pediatrics, Behavioral weight management, Lifestyle treatment, Anti-obesity medication, Metabolic and bariatric surgery

## Abstract

**Purpose of Review:**

Eating disorders among children and adolescents occur in all body sizes and must be properly identified and treated across settings for optimal health outcomes. This review summarizes considerations for behavioral, pharmacological, and surgical obesity treatment and eating disorder risk.

**Recent Findings:**

Obesity and eating disorders share many common risk factors. Yet adolescents with obesity and eating disorders are underrepresented in research studies and often remain undiagnosed in medical practice despite presenting with more severe symptoms. There is evidence for reduction of subclinical disordered eating attitudes and behaviors in health behavior and lifestyle trials, with emerging evidence of reductions in subclinical disordered behaviors (i.e., loss of control eating) for patients who have undergone bariatric surgery. There is a paucity of eating disorder screening and monitoring data reported in pharmacological trials. Psychosocial outcomes, including subclinical eating disordered attitudes and behaviors, suicidality risk, and symptoms of depression and anxiety, should be continuously monitored prior to and throughout obesity treatment. There are several validated measures to detect symptoms of disordered eating among youth with higher weights. Early detection of eating disorder symptoms among obesity treatment-seeking youth is imperative to inform the course of comorbid treatment to occur simultaneously, sequentially, or within specialty care.

**Summary:**

Adolescents with obesity are at greater risk for engaging in disordered eating and related worsened mental and physical health outcomes compared to peers without obesity. It is vital to assess for eating disorder symptoms and presentation to inform timely treatment.

## Introduction

Obesity is a chronic disease associated with harmful physical and mental health complications [1], requiring longitudinal treatment. Over 1 in 5 U.S. children and adolescents between the ages of 2 and 19 have obesity (body mass index, kg/m2, [BMI] ≥ 95th percentile) [2]. The 2023 American Academy of Pediatrics Clinical Practice Guideline for the Treatment of Child Obesity (AAP CPG) recommends treatment as early as possible, as opposed to previous models of a staged approach where providers were advised to increase intensity of treatment dependent on weight change over time [3, 4]. In addition to intensive health behavior and lifestyle treatment (IHBLT), the release of the AAP CPG recommends providers consider pharmacological and surgical treatment for adolescents, dependent on age, availability, and comorbidities [3]. In alignment with these guidelines, the United States Prevention Services Task Force (USPSTF) urges clinicians to be aware of eating disorder risk factors, signs and symptoms, to listen to any patient concerns about eating, and to ensure that youth are referred for treatment as necessary [5]. 

Obesity and eating disorders in youth share several risk factors [6], and in fact, those with obesity exhibit higher rates of eating disorders and subclinical symptoms, compared to peers without obesity [7]. These increased rates are particularly concerning given the adverse mental and physical health outcomes associated with eating disorders [8–11]. To date, there is a lack of routine assessment of youth with eating disorders in obesity treatment studies and in practice settings despite robust evidence of individuals with high body mass index having elevated risk [12, 13]. This gap in research procedures prohibits timely referral for youth who would benefit from specialized treatment.

The purpose of this report is to review the co-occurrence of disordered eating attitudes and behaviors among adolescents seeking obesity treatment. We describe evidence of eating disorder risk and improvements among obesity treatment approaches. Furthermore, updated information is provided on cost- and time-efficient screening and monitoring tools that are practical within pediatric practices that facilitate early intervention and appropriate referral.

## Who Has an Eating Disorder and How Do They Present?

Approximately 28 million, or, 8% of Americans are estimated to develop an eating disorder in their lifetime [14]. These disorders are characterized by persistent disturbances in eating that result in altered consumption or absorption of food that contributes to worsened health [15]. Disorders outlined by the American Psychiatric Association include pica, rumination disorder, avoidant/restrictive food intake disorder, anorexia nervosa, bulimia nervosa, binge-eating disorder and other specified (or unspecified) feeding and eating disorders (Table 1) [15]. Eating disorders are associated with severe physical health complications, including malnutrition, electrolyte imbalances, cardiovascular issues, and potential organ failure, which can be life-threatening [17]. Youth with restrictive eating disorders face additional medical risks including irreversible and deleterious impacts on linear growth, bone deposition, and brain development [18]. Moreover, eating disorders are often comorbid with other mental health conditions, such as depression, anxiety, obsessive-compulsive disorder, attention-deficit/hyperactivity disorder, and substance abuse [19]. These associations are shown to exacerbate psychological distress, potentially leading to a reduced quality of life and elevated suicidality [20]. Early identification of and intervention for eating disorders are critical for improving prognoses [21]. 

**Table 1:** Summary of terms

Term	Characteristics
Anorexia nervosa[15]	• Restriction of energy intake • Intense fear of gaining weight • Disturbance in self-perceived weight or shape
Atypical anorexia nervosa[15]	• All the criteria for anorexia nervosa are met • Despite significant weight loss, the individual’s weight is with or above the healthy range
Avoidant/restrictive feeding or eating disorder[15]	• Eating or feeding disturbance that results in failure to meet appropriate nutritional and/or energy needs • Not due to a lack of available food or culturally sanctioned practice
Binge-eating disorder[15]	• Recurrent episodes of binge eating (i.e., eating an objectively large amount of food in a discrete period of time, with a sense of lack of control over eating) • Marked distress regarding binge eating • Binge eating episodes associated with rapid eating, eating until feeling uncomfortably full, eating large amounts of food despite no physical hunger, eating alone or in secret, feelings of disgust/depression/guilt • No compensatory behavior
Bulimia nervosa[15]	• Recurrent episodes of binge eating (i.e., eating an objectively large amount of food in a discrete period of time, with a sense of lack of control over eating) • Recurrent inappropriate compensatory behaviors (e.g., self-induced vomiting, misuse of laxatives/diuretics, fasting, excessive exercise) • Self-evaluation is unduly influenced by body shape and weight
Health behavior and lifestyle treatment (HBLT)[3, 16]	• Child-focused, family-centred, coordinated approach to care • Coordinated in a medical home and involving pediatricians, registered dietitian nutritionists, mental health providers, nurses, exercise specialists • Integrates components of family, school, community settings and health policy • Takes a chronic, life course approach to identification and treatment, with longitudinal engagement
Intensive health behavior and lifestyle treatment[3]	• Same components as HBLT (whole family engagement, nutrition, physical activity, and behavior change components) • Most effective when 26 h are delivered face-to-face across 3 to 12 months • May be offered as a defined program within pediatrician and other primary care offices, medical centers, health systems, or community programming
Other specified (or unspecified) feeding and eating disorder	• Designed to capture presentations of feeding and eating disorders that cause significant distress or impairment but do not meet the full criteria for any of the specific disorders • Significant distress or impairment is present • Symptoms may be of sufficient severity to warrant clinical attention, distinguishing them from variations in normalized eating behaviors

Eating disorders likely affect a much larger portion of the population than current data indicate, primarily due to underreporting, misdiagnosis, and the pervasive stigma surrounding mental health and body image [22, 23]. An estimated 22% of surveyed youth meet eating disorder criteria, with increased risk for bulimia nervosa, anorexia nervosa, atypical anorexia nervosa, and binge eating disorder among those with elevated BMI [24]. Patterns of subclinical eating disorders are frequently overlooked in public health surveillance, yet still have significant health consequences [25]. The AAP CPG calls for screening for eating disorders prior to implementing treatment for youth at higher weights [3], yet barriers hamper the implementation of these practices. Weight bias and stigma contribute to incomplete and/or absent assessment at medical care visits, where youth with obesity are primarily screened only for binge eating disorder, if screened at all [26]. In a sample of 260 medical providers, 68% of respondents indicated that they did not think to screen for an eating disorder because it was not the presenting concern and nearly 59% did not feel like they had the skills necessary to intervene [27]. A survey of 59 obesity care providers in Australia noted a need for guidance and training on how to screen for and manage eating disorders [28]. Practical guidelines to mitigate risk in youth undergoing obesity management have been proposed including monitoring for unintentional weight reduction, inadequate dietary intake, and stressors that may trigger unhealthy weight control practices and discouraging unhealthy weight talk and skipping of meals [29]. 

### Restrictive Eating Disorders

Restrictive eating disorders can occur at higher body weight and with serious complications [30]. Restrictive eating disorders include atypical anorexia nervosa, characterized by significant distress and rapid weight loss where the person’s weight falls within healthy or higher ranges, and avoidant/restrictive food intake disorder, characterized by highly selective eating habits. To better understand the relationship between obesity and eating disorders characterized by weight reduction and/or dietary restriction, researchers conducted a retrospective cohort study on patients diagnosed with either anorexia nervosa or eating disorder not otherwise specified (characterized by weight loss and/or dietary restriction) [31]. Of 179 adolescents, 36.7% had a BMI history above the 85th percentile. These patients had a larger BMI decrease at presentation and longer illness duration before presentation than for those with a lower BMI percentile, suggesting a more severe profile. Thus, youth with a history of overweight or obesity represent a substantial portion of treatment-seeking adolescents with restrictive eating disorders, underscoring that rapid and extreme weight reduction in adolescents is concerning (i.e., >10% across 3–6 months, >1 kg/week across 6 weeks), regardless of whether the end weight is theoretically within or above a healthy range [32, 33]. Youth with avoidant restrictive food intake disorder (ARFID) are up to five times more likely to have higher frequency of elevated cholesterol compared to healthy control peers, and more than one-third of youth with ARFID report gastrointestinal and immune comorbidities [34]. Nutrient deficiencies are evident among youth with ARFID with higher weight that should be monitored and treated intensively given the damaging effects of malnutrition on growth and development [18]. 

### Binge Type Eating Disorders

Binge-eating disorder is estimated to occur in between one and five% of youth, with a recent systematic review of 73,164 participants under age 20 identifying binge eating disorder among 1.3% of participants [35]. This disorder is characterized by consuming an objectively large amount of food in a short amount of time with the feeling of a loss of control while eating. Higher rates of binge eating and loss of control eating that do not reach criteria for an eating disorder (i.e., subclinical) are highly prevalent, estimated at 26.3% in children and adolescents with overweight and obesity [36]. The global prevalence of bulimia nervosa in children and adolescents is estimated to be approximately 6% as of 2019 [37]. Binge-eating disorder, binge eating, and loss of control eating are each associated with BMI and increased psychiatric impairment [38]. 

## Shared Risk Factors

There are many shared risk factors between obesity and eating disorders [39–43]. Behaviors which predict eating disorder development among all body sizes include self-directed dieting (SDD), early binge and loss of control eating, and over-valuation of body shape and weight [44, 45]. Experiences with discrimination place certain individuals with social disadvantage at greatest risk, especially if they experience multiple marginalization [46]. Youth with obesity are exposed to weight bias and stigmatization (i.e., negative attitudes, beliefs, and behaviors based on body size) and report more experiences with weight-related teasing and critical appearance-based comments from friends and family compared to peers with lower weight. Bias and stigma exacerbate body image concerns, unhealthy weight control efforts, patterns of loss of control eating, and physiological activation of pathways involved with metabolism and inflammation [47]. In turn, these effects contribute to increased weight and continued subjection of bias and stigma, thus underlying the complex nature of weight bias and stigma, higher weight, and disordered eating. Heightened awareness and understanding of eating disorder risk amongst diverse body types can facilitate prompt intervention, thereby mitigating the disorder’s duration and severity. Given well-documented overlap in symptoms of heightened depression, anxiety, food insecurity [48], and stigma [49], providers should assess for these factors as they are related to effective treatment for both obesity and disordered eating [43, 50]. 

## Dieting vs. Evidence-Based Behavioral Treatment

SDD and evidence-based obesity treatment often share the same goal – weight reduction or stabilization. However, SDD increases a wide array of weight control behaviors, ranging from healthy behaviors that are consistent with the Dietary Guidelines for Americans [51] (e.g., reducing added sugar, increased fruit and vegetables) to unhealthy and extreme behaviors (e.g., fasting), some of which (e.g., vomiting, laxative pills) are disordered [52]. In a sample of 412 adolescents with obesity 31% endorsed disordered eating/unhealthy weight control practices in SDD attempts. Of those, 86% reported using at least one extreme weight control behavior (e.g., using laxatives or diet pills) while 32% reported binge eating with loss of control [53]. The majority of youth however (69%) engaged in healthier eating practices (e.g., ate more fruits and vegetables, reduced high-fat intake). A similar population level surveillance of adolescent weight control behaviors by Brown and colleagues found the majority of sampled youth (*n* = 6,117) ages 8–15 years attempted to reduce weight via positive strategies such as reducing high-fat and sugar intake (84%); however, some endorsed concerning behaviors such as skipping meals (35%), and extreme restriction (18%) [54]. 

Observational evidence links SDD with increased risk for disordered eating [55–57] and is predictive of excess weight gain and obesity [58]. When SDD is examined by type of behavior, the use of unhealthy and extremely restrictive weight control behaviors (e.g., skipping meals, excessive exercise) appears to increase disordered eating risk for some individuals [55, 56]. Notably, SDD that incorporates moderate caloric reduction and that is less rigidly associated with appearance can result in positive weight related outcomes and psychological benefits (e.g., reduced binge eating without the development of disordered eating) [59, 60]. Evidence-based health behavior and lifestyle treatment (HBLT) consistently decreases risk of eating disorders in both children [16] and adults [61]. HBLT is characterized by a child-focused, family-centered, coordinated approach to care with medical oversight [3]. Reducing total energy intake/dietary restraint via flexible self-monitoring and goal setting is a cornerstone of HBLT. In the context of an obesogenic environment, this type of restraint may be beneficial [45]. Indeed, HBLT focuses on increasing sustainable health-promoting behaviors (e.g., regular meal patterns) to support overall health, as opposed to focusing primarily on weight. Moreover, behavior change strategies can be successfully implemented to improve dietary quality while limiting restriction while supervised treatment also allows for monitoring of potential disordered eating [1]. 

The distinction between dieting and obesity treatment has been illustrated in the psych-behavioral dieting paradigm by Haynos and colleagues [62]. In this paradigm, there are two dimensions of dieting behavior – behavioral and psychological. The behavior dimension (high/low) consists of the weight control behaviors used, ranging from moderate efforts such as limiting portions (low), to extreme efforts, such as fasting (high). The psychological dimension (positive/negative) focuses on the emotional, cognitive, and motivational indices behind the behavior including rigid adherence practices and extreme dieting goals (negative) to approaching dieting flexibly and focusing on health-related goals (positive). Within this paradigm, HBLT which is characterized by lower behavioral demands and more positive psychological dimensions (i.e., moderate, flexible) stands in contrast to typical SDD, which tends to involve higher behavioral demands and more negative psychological dimensions (i.e., extreme, rigid). Individuals who participate in HBLT are guided to develop a positive psychological approach by setting moderate and achievable goals regarding diet in combination with efforts low on the behavioral index [1]. On the other hand, individuals using a SDD approach may inadvertently identify a more negative thinking pattern or adoption of extreme behaviors depending on intrapersonal factors and available resources.

## Eating Disorder Risk and Outcomes in Health Behavior and Lifestyle Treatment

IHBLTs are endorsed [3, 63, 64] as the first line of treatment for youth with obesity. IHBLTs incorporate education and support to improve diet quality, increase physical activity, and reduce sedentary behaviors using a range of behavior change techniques to build skills in problem-solving and goal setting [1]. In family-based treatment the responsibilities for parents are emphasized in serving as role models and as change agents within the home environment. IHBLT differs from HBLT in that it is most effective when 26 h of face-to-face contact delivered across 3-to-12-months. Reviews evaluating effects of HBLT share similar principles, but IHBLT thresholds were identified by the AAP CPG in 2023 [1]. The effects of HBLT on subclinical disordered eating behaviors, and a range of psychosocial factors associated with the development of eating disorders, have been examined. Table 2 summarizes systematic reviews and meta-analyses reporting on eating disorder risk and related factors [16, 38, 65–76]. Current literature suggests that subclinical eating disorder symptoms are reduced in HBLT, however, it is important to note that individuals diagnosed with eating disorders are often screened out which may limit the generalizability of these findings for adolescents in the real-world who require more specialized treatment.

**Table 2:** Summary of results from systematic reviews assessing eating disorder risk and related risk factors in children (ages 4-12y) and adolescents (ages 13-19y) participating in obesity treatments

Review citation	Study characteristics	Outcomes	Follow-up ranges
Eating disorder symptoms and eating behaviors			
Butryn and Wadden 2005[65]	Behavioural interventions 5 studies	No increase in eating disorder symptoms with some improvements in psychological status.^a^	6–10 years
De Giuseppe, R., et al. 2019[66]	Behavioural interventions 9 studies	Significant decrease in external and emotional eating, 3 studies. Significant increase in restraint eating, 4 studies. Significant decrease of binge eating symptoms, 2 studies. Improvement of self-perception and shape concern, 2 studies.^a^	Up to 5 years
Jebeile et al., 2019[16]	Behavioural interventions 30 studies RCTs and pre-post studies	Reduced prevalence of some disordered eating symptoms. **Meta-analysis**,** within-group change**,** post-intervention** Global eating disorder risk, 9 studies SMD [SE] − 0.10 [0.10], *p* = 0.317 Bulimic symptoms, 8 studies, − 0.326 [0.09], *p* < 0.001) Emotional eating, 6 studies, − 0.149 [0.06], *p* = 0.008 Eating concern, 4 studies, − 0.19 [0.13], *p* = 0.13 Binge eating, 3 studies, − 0.588 [0.10], *p* < 0.001 Drive for thinness, 3 studies, − 0.167 [0.06], *p* = 0.005 **Meta-analysis**,** within-group change**,** follow-up** Global eating disorder risk, 6 studies, −0.313 [0.13], *p* = 0.012 Bulimic symptoms, 3 studies, − 0.25 [0.24], *p* = 0.30 Emotional eating, 5 studies, − 0.259 [0.05], *p* < 0.001 Eating concern, 3 studies, − 0.501 [0.06], *p* < 0.001 Drive for thinness, 2 studies, − 0.375 [0.07], *p* < 0.001	6 months- 6 years
Moustafa et al. 2021[38]	Behavioural interventions, pharmacotherapy, and surgical interventions 29 studies RCTs, case-control, longitudinal cohort studies	BE/LOC behaviours significantly decreased following interventions in 20 of 21 studies.^a^ A greater decrease in BE/LOC was associated with improved weight reduction in 4 of 9 studies that assessed this change. The remaining 5/9 studies found no significant change [67–69].	6 months- 6 years
House et al. 2021[67]	Behavioural interventions 23 studies RCTs and pre-post studies	Dietary restraint increased (7/20 studies; no changes 13/20) while dieting showed no (5/8 studies), decreased (2/8 studies), and increased (1/8) changes.^a^ In the same studies, other eating disorder risk factors (e.g., binge eating [3/3], body dissatisfaction [6/10], and depression [2/6]) were improved or unchanged [0/3, 4/10, 4/6, respectively] at postintervention and/or follow-up.	10 weeks-6 years
Colombo et al. 2024[68]	Behavioural interventions 8 studies RCTs	**Meta-analysis**,** between intervention and standard care/no-treatment control** **Post-intervention**,** favours intervention that increase dietary restraint** Dietary restraint, 3 studies, SMD 0.42 [95%CI 0.13, 0.70] **Follow-up**,** no difference between groups** Dietary restraint, 3 studies, SMD 0.16 [95% CI 0.28, 0.60] There were no differences between intervention and control for most eating behaviours. Improvements in emotional eating, external eating, food responsiveness, and enjoyment of food were reported in individual studies.	12–52 weeks
Depression and anxiety			
Herget et al. 2014[69]	Bariatric surgery 6 studies Retrospective chart review, pre-post studies	Improvements of mean levels of depressive symptoms were observed in all studies (7/7).^a^	6 months-9 years
Hillstrom et al. 2015[70]	Bariatric surgery Studies 5 studies RCT and prospective studies	Articles (6/6) reported a significant improvement (decrease) in depression by most patients.^a^	4 months-11.5 years
Jebeile et al. 2019[16]	Behavioural interventions 44 studies (36 studies, depression; 10 studies, anxiety) RCTs and pre-post studies	**Meta-analysis**,** within-group change** **Depression** Reduced depressive symptoms postintervention, SMD [SE], − 0.31 [0.04]; *p* < 0.001). Follow-up, 11 studies, − 0.25 [0.07]; *p* < 0.001. **Anxiety** Reduced anxiety post-intervention, SMD [SE], − 0.38 [0.10]; *p* < 0.001) Follow-up, 4 studies, − 0.32 [0.15]; *p* = 0.03	6 months-6 years
King et al. 2020[71]	Behavioural interventions with moderate to vigorous physical activity 10 studies RCTs and pre-post studies	**Meta-analysis**,** within-group change** Reduced depression, SMD [SE] − 0.34 [0.06], *p* < 0.001	7 weeks-2.4 years
O’Connor et al. 2024[72]	Review of interventions for high body mass index in children and adolescents	No changes in depression scores for 3/3 behavioral intervention studies. Compared to 2.4% of participants in the control group, 4% of those receiving liraglutide developed depression in 1 study. *P* value not reported.	13–16 months
Self-esteem			
Murray et al. 2017[73]	Behavioural interventions 13 studies RCTs	**Meta-analysis of 13 studies up to the year 2014**,** intervention vs. control** No significant change in self-esteem between intervention and control (0.27 [−0.04, 0.59]). Within-intervention arms, 3 studies showed improved self-esteem and 5 studies showed no effect. There were no positive effects on self-esteem for any control arms, one wait-list control group showed a small negative effect on self-esteem. Interventions may have been too general to address this construct.	28 weeks-2 years
Gow et al. 2020[74]	Behavioural interventions 49 studies RCTs and pre-post studies	**Meta-analysis of 49 studies up to the year 2019**,** within-group change** Increased self-esteem post-intervention SMD [SE] 0.34 [0.03], *p* < 0 0.001 Follow-up, 17 studies SMD [SE] 0.35 [0.05] *p* < 0.001	2 weeks-6 years
King et al. 2020[71]	Behavioural interventions with moderate to vigorous physical activity 21 studies RCTs and pre-post studies	**Meta-analysis**,** within-group change** Increased self-esteem, SMD [SE] 0.34 [0.04], *p* < 0.001	7 weeks-2.4 years
Body image			
Gow et al. 2020[74]	Behavioural interventions 40 studies RCTs and pre-post studies	**Meta-analysis**,** within-group change** Improved body image post-intervention SMD [SE] 0.40 [0.03], *P* < 0.001 Follow-up, 16 studies SMD [SE] 0.41 [0.08], *P* < 0.001	2 weeks-6 years
King et al. 2020[71]	Behavioural interventions with moderate to vigorous physical activity 17 studies RCTs and pre-post studies	**Meta-analysis**,** within-group change** Improved body image, SMD [SE] 0.47 [0.05], *p* < 0.001	7 weeks-2.4 years
Quality of life			
Murray et al. 2019[75]	Behavioural interventions 8 studies RCTs	**Meta-analysis**,** intervention vs. control** Improved quality of life Mean difference 0.20 [0.11, 0.29]; *p <* 0.01	28 weeks-2 years
Hillstrom et al. 2015[70]	Bariatric surgery Studies 9 studies RCT and prospective studies	Improved quality of life for most participants across several domains.^a^	4 months-11.5 years
Skinner et al. 2023[1]	Behavioral intervention studies (215) on obesity outcomes	Improved quality of life for 8/19 studies, no change in 11/19.^a^	6 weeks-2 years
O’Connor et al. 2024[72]	Review of interventions for high body mass index in children and adolescents	Small, non-significant, increases in total quality of life in behavioral interventions < 26 h. Between group differences show improvement for 3/11 studies. Mean difference 1.9 [0.2–3.5], *p* = 0.17 No significant differences for > 26 h. Increased weight-related quality of life with semaglutide in 1 study. Mean difference 5.3 [0.2–8.3]. No *p* value reported. Remaining 3/4 pharmacotherapy studies found no differences.	13–16 months

*SMD*, standardized mean difference; *SE* standard error

^a^SMD, SE, *p* values not reported

Individuals diagnosed with eating disorders are regularly screened out of behavioral trials, however, current literature suggests that subclinical eating disorder symptoms and behaviors are reduced following HBLT [16, 66]. Recent trials also support findings from these reviews, for example, an effectiveness trial of two pediatric weight management interventions for 407 children aged 6–12 years, reported a decreased prevalence of sneaking, hiding and hoarding food in all participants from 29.1% to 20.7% at 12 months [77]. Eating in the absence of hunger also decreased in all participants from 46.7% to 22.4% at 12 months [77]. Similarly, a trial was conducted with 172 parent–child dyads and provided 4-months of family-based treatment followed by a maintenance interventions [44]. Reduced shape and weight concern was reported following the family-based treatment and maintained at follow-up [44]. Another trial comparing family-based treatment with parent-only treatment for 150 children, 8–12 years found reductions in shape, weight, and eating concerns at the end of treatment and reduced loss of control over eating post-treatment and follow-up [78]. Finally, an intervention comparing two dietary interventions alongside HBLT in 141 adolescents with obesity, reported reductions in eating disorder risk scores in both groups [79]. In this trial reduced binge-eating severity was seen in both groups following a 4-week very low energy diet, but was only maintained at 12 months in the group following intermittent energy restriction but not in the group following continuous energy restriction [79]. 

Family-based treatments targeting the individual and socio-environmental components of weight management overlap with treatments for disordered eating and eating disorders (e.g., regular eating, regular weigh-ins, improving dietary quality) [49]. These shared components may account for the improvement in subclinical eating disorder symptoms and behaviors seen in the aforementioned trials. Given long-term associations of eating disorders with later depression, anxiety, substance use, and obesity [80], weight management interventions may play a pivotal role in stabilizing or reducing eating disorder risk at this critical time [81, 82]. Although positive outcomes are seen for most participants in HBLT a subset of youth may present with a previously undiagnosed eating disorder or may develop an eating disorder during the course of treatment. Current evidence suggests that 5–10% of individuals participating in behavioral interventions may require additional support for disordered eating [79, 83]. However, this is based on limited data from few trials and with no causal relationships reported. Longer term data and larger trial data sets are needed to understand, phenotype, and prevent such risk in the future [84]. Ongoing monitoring of flexible and moderate restraint with regular patterns of eating would likely be beneficial in these trials.

## Eating Disorder Risk and Outcomes in Pharmacological Obesity Treatment

There are currently 4 U.S. FDA-approved medications for obesity treatment available to adolescents ages 12+: Orlistat [85], Wegovy [86], Saxenda [87], and Qsymia [88]. Certain medications are glucagon-like peptide-1 receptor agonists (GLP-1 RA; i.e., Wegovy, Saxenda), but not all (i.e., Xenical, Qsymia; lipase inhibitor). GLP-1 RA treatments (and Qsymia) work by increasing satiety signaling (and for some, slowed gastric emptying), while other mechanisms of action include the prevention of fat absorption (Xenical, lipase inhibitors), contributing to significant weight reduction. Pharmacotherapy agents have also demonstrated early success with improvements in markers of cardiometabolic health and quality of life [89]. 

Given the relatively new recommendation for clinical use of pharmacotherapy for pediatric obesity treatment [3] and limited long-term follow up trials, there is a dearth of data available to identify and determine psychological and behavioral outcomes associated with pharmacotherapy treatment, in particular disordered eating [12]. Pharmacotherapy trials attempt to exclude patients with eating disorders, yet assessment procedures are inconsistent and insufficient. One study sought to screen out patients with diagnosed eating disorders, however it did so by excluding patients with weight reduction, psychiatric disease, or a history of “bulimia”, “laxative abuse” or “anorexiants” [85]. These described conditions do not meet the scope of eating disorders as currently understood [15]. Another study excluded adolescents with a diagnosis of bulimia nervosa. However other eating disorders were not excluded nor was any eating disorder screening described [90]. Absence of broad eating disorder screening may be driven by limited understanding of the breadth of eating disorders seen in youth at higher weights and the unavailability of validated eating disorders screening tool for this age group.

Given the mechanisms of action of pharmacological treatment for obesity that reduce intake, adolescents are likely to experience rapid BMI reduction [91]. While research shows significant BMI reductions, the effects of rapid weight loss such as disordered eating symptoms remain unclear. Of concern are health risks from extreme restriction, similar to atypical anorexia nervosa, which may go undetected in settings where weight reduction is a treatment goal. Malnutrition in atypical anorexia nervosa, as described in non-pharmacological studies, involves deficiencies in energy, protein, or micronutrients which can severely impact growth and development [33]. The imbalance between nutrient supply and demand is critical in youth, leading to acute and chronic malnutrition [33]. Acute malnutrition often manifests as weight reduction, while chronic malnutrition can result in stunted growth, which may have long-term and potentially irreversible effects [33]. Guidelines for nutritional recommendations for youth using obesity pharmacotherapy treatment are not yet available, but monitoring and guidance by trained behavioral and nutritional health providers regarding adequate nutrition in light of restricted intake is critical [92]. 

Rate and magnitude of weight reduction have been found to predict serious medical complications in patients with restrictive eating disorders. Complications include bradycardia, hypotension, electrolyte disturbances, low estrogen/testosterone levels, and low bone mineral density, and are seen in youth as well as adults [18, 93]. Nearly 20% of patients admitted for acute medical stabilization due to eating disorders have atypical anorexia nervosa, highlighting the seriousness of the condition [34]. For these reasons the AAP cautions providers to screen for “unhealthy and extreme weight-control measures before praising desirable weight loss.” [3] Although not intended to be an unhealthy weight control measure, pharmacotherapy may pose risk for development or exacerbation of disordered eating behaviors with ensuing serious medical complications, however more data is needed to understand the magnitude of this risk.

## Eating Disorder Risk and Outcomes in Surgical Obesity Treatment

Bariatric surgery is associated with positive effects for weight reduction, weight-related quality of life [94], self-esteem, mood, and decreases in anxiety and depression [95]. However, the positive effects of these associations may decrease overtime, requiring ongoing clinical treatment [69]. Among youth seeking surgery, common eating disorder diagnoses include binge-eating disorder, night eating syndrome, bulimia nervosa, and other specified feeding or eating disorder [96]. Research suggests that rates of subclinical disordered eating among those seeking surgery may be similar across gender and race (female, White) in adolescence, although it’s unknown if diverse populations seeking surgery are also engaging in disordered eating at similar rates given the lack of studies and small samples [96]. 

Youth preparing for surgery are reported to endorse greater symptoms of subclinical disordered eating across multiple domains, compared to those participating in HBLT [97]. Importantly, eating disorders are not contraindications for surgery, but should be stabilized and treated both before and after to the extent possible [98]. Significant reductions in binge type eating are evident post-operatively, as well as greater reductions in weight and shape concerns [99]. As described by Reddy and colleagues [100], anatomical changes following surgery limit the amount of food that an individual can consume, thus, reducing rates of binge-eating disorder and objective binge-eating in the months following the procedure. Goldschmidt and colleagues determined that preoperative loss of control eating was not associated with BMI change post-operatively, but the development of this behavior was associated with lower percent BMI change at 1, 2, and 3-year follow-ups [101]. However, among adult samples, it is suggested to assess for and treat disordered eating prior to metabolic surgery, as binge-eating disorder is consistently predictive of poor weight reduction outcomes [102]. Further, adults at risk for an eating disorder or who experience loss of control eating post-surgery lose significantly less weight and have higher scores in internalizing behaviors (e.g., emotional dysfunction, low positive emotions) compared to those with minimal to no risk for eating disorders. Thus, postoperative disordered eating symptoms are related to and may be exacerbated by greater experience of negative emotions after surgery [103]. 

As with pharmacotherapy, behavioral and nutritional monitoring before and after treatment is essential to ensure adequate nutrition during rapid weight reduction [98]. Bariatric surgery carries risks such as macro- and micronutrient deficiencies due to reduced food intake similar to what is seen in atypical anorexia nervosa and limited fat absorption [98]. Although atypical anorexia nervosa has been reported at low rates in adults following bariatric surgery [104], these cases have not been documented in youth populations.

### Recommendations

The generalizability of obesity treatment trials eating disorder risk outcomes are limited due to inconsistent assessment, monitoring, and inclusion criteria, as noted in the USPSTF report on Screening for Eating Disorders [105]. Researchers may benefit from a better understanding of which clinical and subclinical disordered eating behaviors are exclusionary (e.g., atypical anorexia nervosa) within obesity treatment trials, and which may benefit with combined pharmacotherapy and HBLT (e.g., binge type eating). Finally, there is a need for researchers to identify behavioral strategies appropriate to support those using pharmacological obesity treatment, as established in bariatric surgery approaches, to optimize non-weight outcomes as these supports may look different compared to HBLT treatment alone [92]. 

Assessment and monitoring of disordered eating and malnutrition throughout obesity treatment research trials and clinical practices are essential, as presence of these symptoms warrant attention and treatment. Validated eating disorder risk screening and ongoing monitoring measures for youth in the context of obesity treatment include the Youth Eating Disorder Examination Questionnaire Brief [106, 107], Questionnaire on Eating and Weight Patterns [108], Adolescent Binge-Eating Questionnaire [109], the Stanford-Washington University Eating Disorders Screen for Youth (SWED) [110], among others [111]. Furthermore, the fields of eating disorders and obesity medicine are coming together to move the needle on effective treatment. Following the AAP CPG, members of the AAP, Academy for Eating Disorders, and representatives from National Eating Disorder Association among others have convened to develop a screening measure to detect eating disorders in youth across the age, gender, weight and behavior spectrum, (Screening Kids’ And Teens’ Eating) currently in the validation stage. This tool could be useful in detecting eating disorders in youth seeking weight management treatment.

Like obesity, eating disorders are chronic and ongoing treatments are necessary. Youth who screen positively for eating disordered thoughts and behaviors should be referred for further assessment and evidence-based treatment. These approaches include enhanced cognitive behavioral therapy, family-based treatment, and interpersonal psychotherapy, with emerging evidence for other approaches (e.g., dialectical behavior therapy, acceptance and commitment therapy, mindfulness-based interventions) [112]. The development and evaluation of integrated treatments for both conditions, through coordinated, patient-centered care models, may be beneficial to address shared risk factors, high comorbidities, and gaps in care that may overlook either eating disorder risk or metabolic health.

## Conclusions

Youth with overweight and obesity are at high risk for having disordered attitudes and engaging in disordered eating behaviors associated with significantly worsened mental and physical health outcomes. Evidence-based HBLT and bariatric surgery are effective in this age group for reducing some aspects of subclinical eating disorder behaviors, with promising benefits found in pharmacological approaches. Given the high rates of comorbidity, the exclusion of individuals with eating disorders from obesity treatment trials limits the generalizability of findings. Efforts and reporting of screening and continuous monitoring of disordered eating symptoms throughout obesity treatment is imperative to determine the development and exacerbation of symptoms and to provide appropriate referrals as needed.

## Key References

- Hampl SE, Hassink SG, Skinner AC, et al. Clinical practice guideline for the evaluation and treatment of children and adolescents with obesity. Pediatr. 2023;151(2)doi:10.1542/peds.2022-060640.
  - A report following systematic evaluation of evidence for child obesity treatment to inform pediatric clinical practice guidance. This report recommends providers offer behavioral, pharmacological, and surgical obesity treatment to child and adolescent patients depending on age and comorbid conditions.
- Tanner AB. Unique considerations for the medical care of restrictive eating disorders in children and young adolescents. J Eat Disord. 2023;11(1):33.
  - This review describes the medical complications related to energy needs and pubertal development for children and adolescents with restrictive eating.
- Garcia Moreno N, Walker DC, Gullo N, O’Dea CJ. Weight stigma’s effects on misdiagnosis of eating disorders among laypeople and healthcare professionals. Int J Eat Disord. 2025.
  - This study found that lay people and health care providers under recognize and under diagnose eating disorders among those with diverse body sizes.
- Hayes JF, Fitzsimmons-Craft EE, Karam AM, Jakubiak J, Brown ML, Wilfley DE. Disordered eating attitudes and behaviors in youth overweight and obesity: Implications for treatment. Curr Obes Rep. 2018; 7:235–246.
  - This review describes the overlapping individual and environmental correlates of obesity and disordered eating among youth.
  - Evidence-based behavioral treatment is designed to address these overlapping components and is shown to reduce both weight and psychosocial challenges.
- Cardel MI, Newsome FA, Pearl RL, et al. Patient-centered care for obesity: how health care providers can treat obesity while actively addressing weight stigma and eating disorder risk. JAND. 2022;122(6):1089–1098.
  - This review describes the differences between self-directed dieting and supervised obesity treatment as well as the overlap between eating disorder and obesity treatment goals and approaches.
- Jebeile H, Gow ML, Baur LA, Garnett SP, Paxton SJ, Lister NB. Treatment of obesity, with a dietary component, and eating disorder risk in children and adolescents: A systematic review with meta-analysis. Obes Rev. 2019;20(9):1287–1298. doi:10.1111/obr.12866.
  - This review highlights the role of behavioral obesity treatment in reducing eating disorder risk, prevalence, and symptoms among youth.
- Nicholson WK, Silverstein M, Wong JB, et al. Interventions for high body mass index in children and adolescents: US Preventive Services Task Force recommendation statement. JAMA. 2024;332(3):226–232.
  - USPSTF evaluation of evidence-based practice effectiveness for child obesity treatment. This committee highly recommends the implementation of intensive behavioral treatment.
- Armstrong SC, Eneli I, Osganian SK, Wagner BE, Waldrop SW, Kelly AS. Pediatric obesity pharmacotherapy: State of the science, research gaps, and opportunities. Pediatr. 2024;154(5).
  - This report describes clinical trial design, guidance of use of pharmacotherapy, and treatment outcomes for child obesity treatment based on leading scientific and clinical expert input, with contributions from adolescent patients and caregivers living with obesity.

## Data Availability

No datasets were generated or analysed during the current study.

## Declarations

### Disclaimer

The opinions and assertions expressed herein are those of the authors and are not to be construed as reflecting the views of the Uniformed Services University or the United States Department of Defense. The project described was supported by Grant Number T32MH019960 from the National Institute of Mental Health. The content is solely the responsibility of the authors and does not necessarily represent the official views of the National Institute of Mental Health or the National Institutes of Health.
